# Processing, Properties, Modifications, and Environmental Impact of Nanocellulose/Biopolymer Composites: A Review

**DOI:** 10.3390/polym15051219

**Published:** 2023-02-28

**Authors:** Elizabeth Aigaje, Ariel Riofrio, Haci Baykara

**Affiliations:** 1 Facultad de Ingeniería Mecánica y Ciencias de la Producción, Escuela Superior Politécnica del Litoral, ESPOL, Campus Gustavo Galindo, Km 30.5 Vía Perimetral, Guayaquil 090506, Ecuador; 2 Center of Nanotechnology Research and Development (CIDNA), Escuela Superior Politécnica del Litoral, ESPOL, Campus Gustavo Galindo, Km 30.5 Vía Perimetral, Guayaquil 090506, Ecuador

**Keywords:** biocomposite, biopolymer, environmental impact, nanocellulose, sustainable materials

## Abstract

The increasing concerns about plastic pollution and climate change have encouraged research into bioderived and biodegradable materials. Much attention has been focused on nanocellulose due to its abundance, biodegradability, and excellent mechanical properties. Nanocellulose-based biocomposites are a viable option to fabricate functional and sustainable materials for important engineering applications. This review addresses the most recent advances in composites, with a particular focus on biopolymer matrices such as starch, chitosan, polylactic acid, and polyvinyl alcohol. Additionally, the effects of the processing methods, the influence of additives, and the outturn of nanocellulose surface modification on the biocomposite’s properties are outlined in detail. Moreover, the change in the composites’ morphological, mechanical, and other physiochemical properties due to reinforcement loading is reviewed. Further, mechanical strength, thermal resistance, and the oxygen–water vapor barrier properties are enhanced with the incorporation of nanocellulose into biopolymer matrices. Furthermore, the life cycle assessment of nanocellulose and composites were considered to analyze their environmental profile. The sustainability of this alternative material is compared through different preparation routes and options.

## 1. Introduction

Sustainable development has emerged as a crucial aspect of developing greener materials. The utilization of fossil fuels in the production of products has had a significant impact on the environment [1]. Hence, there is a growing demand for products manufactured using renewable and sustainable raw materials instead of petroleum. The significance of this lies in the fact that the United States Department of Energy (DOE) predicts that by 2050, approximately 50% of chemical building blocks will come from renewable sources [2]. As a result, the focus has shifted towards natural polymers, be they animal- or plant-based materials. Among the most readily available natural polymers, cellulose is the most abundant and is found in wood and plants as a composite material along with hemicellulose, lignin, and waxes. Cellulose has several unique characteristics, including being biodegradable, nontoxic, and renewable [3] Additionally, there has been an increase in research on green composites, which can consist of different natural fibers or cellulose-containing materials [4,5,6,7]. Natural fibers and biodegradable polymer composites, such as those made from PLA or starch with cellulose at varying sizes, are also being studied [8,9].

**Figure 1:**
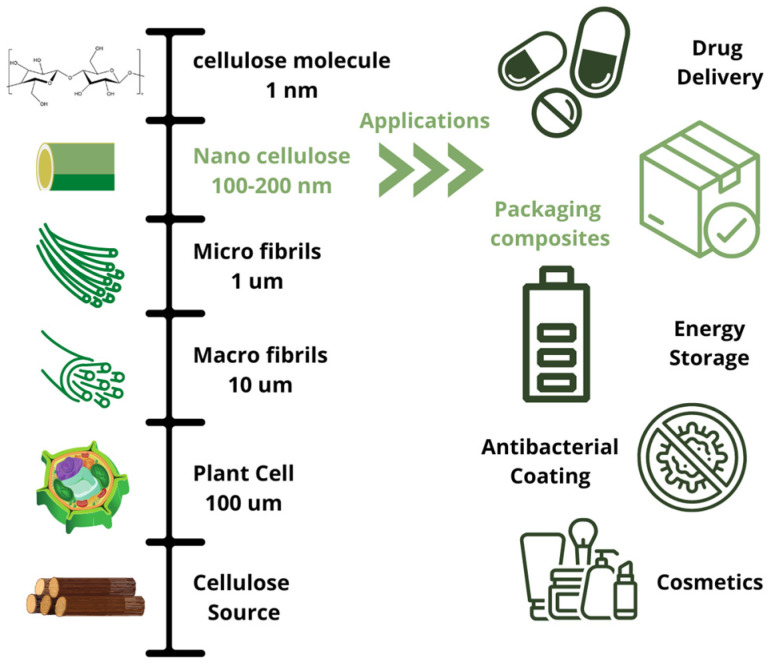
Nanocellulose and its applications in different industries.

Nanocellulose (NC) can be obtained from different sources that contain cellulose [10,11]. Cellulose microfibrils have a cross section in the range of 20–100 nm and a length of 100–200 nm in size. The amorphous part of the cellulose can be easily broken with mechanical treatment. Meanwhile, the crystalline part is much harder to break up. The process involves mechanical, chemical, and physical methods to produce crystals or fibrils of nanocellulose [12]. NC has numerous applications, especially in electronics, energy storage, catalysis, packaging materials, remediation of environmental burdens, drugs and drug delivery, tissue engineering, aerospace engineering, automobile design, and more [9]. Figure 1 depicts the scale from wood to nanocellulose and some of the different applications for NC. NC can be used as filler in a polymer matrix with a positive impact on the mechanical properties in contrast to microsize fillers [13,14]. Synthetic polymers can be replaced by the use of biocomposites that use NCs [15]. Biopolymer has an abundant source of cellulose and has a low density and great mechanical strength that might result in the better performance of composites [16]. NC has an important elasticity modulus (max 150 GPa), a coefficient of thermal expansion with a low score (0.01 ppm/K), and a large surface area. These properties make it interesting as a reinforcement even with a low filler content [17]. However, the properties of the composites will depend greatly on the polymer matrix, the type of nanocellulose, the dispersion of the filler, and the interaction between the nanocellulose and the matrix [18,19]. The biodegradability of the composite has shown the importance of considering the polymers obtained from nature over synthetic ones. Nevertheless, thermal stability can affect how nanocellulose is produced at higher volumes. More research on the effect of temperature on processing may clear any unknown areas in the production of NC [15].

Polymeric composites of NC may be classified depending on the type of polymer used as a matrix. Biopolymeric matrices with NC can result in a composite with better mechanical properties and enhanced qualities. Nanocellulose used as a filler in a polysaccharide matrix in biopolymers such as starch or chitosan has been extensively studied [20,21]. Other polymer matrices found in the literature are polylactic acid (PLA) and polyvinyl alcohol (PVA) [22,23]. Solvent casting and melt processing are the two most common techniques that are used for polymer composite preparation. Mechanical and other properties can be affected depending on the type of process applied [24,25]. Other factors that affect composite properties are modifications on the NC filler, particle size, aspect ratio, and source of cellulose [26,27,28].

Despite the method of preparation or the polymer matrix, the sustainability of new composites should be evaluated to compare available options with innovations. Life cycle assessment (LCA) is a practical methodology that can be applied when producing different alternatives. It is based on the international normative ISO 14040 [29], and it assesses the whole life of a product or service considering the production of the raw material, the processing, the use, and the disposal phase. For this reason, the LCA can be applied to polymers and polymer composites to verify the sustainability of the option [30]. The environmental impact of producing nanocellulose, in its different forms, has been evaluated in the literature [31]. However, only a few studies were found addressing the burden of nanocellulose composites [32].

Several review papers were published in 2020 that explored the development of nanocellulose-reinforced composites using biopolymers as matrices [33,34]. Additionally, review articles published between 2021 and 2022 have summarized the properties of nanocellulose composites in starch and PLA matrices [35,36], the chemical modification methods for the preparation of nanocomposites [37], the development of nanocellulose biobased composites for wastewater remediation [38,39], and drug delivery applications [40]. This study provides an updated and comprehensive analysis of research on nanocellulose composites prepared using biodegradable polymer matrices. It also includes a life cycle assessment of the production of nanocellulose and nanocellulose biobased composites, offering a more comprehensive understanding of the development of ecofriendly nanocellulose composites. The articles included in this study were published in the last two years and have not been included in any other review articles at the time of writing.

The aim of this study is to present a thorough analysis of the various composites found in the literature that use nanocellulose (NC) as a filler in a polymeric matrix. Figure 1, shown in green, illustrates the applications of NC reviewed, focusing on composites using biopolymers as matrices and NCs as filler, primarily in packaging. This review paper collects literature on various production techniques in NC composites, the effect of additives, the composites’ morphology, the mechanical properties with the addition of NC, thermal behavior, oxygen and water vapor transmission properties, optical properties, and the biopolymeric materials’ degradability. In addition, modifications to NC and the environmental impacts of NC and composites are also covered in the review.

## 2. Methodology

The articles collected in this review were found using the SCOPUS database taking only the articles published in 2021 and 2022 into account. A total of 71 articles were considered using the keywords “nanocellulose” and “reinforcement” and then limiting the results to articles that contain the “biocomposites” keyword. Moreover, 259 documents were found using the keywords “cellulose nanocrystals” and “reinforcement,” which then were refined to 77 documents using the keyword “biopolymer”. Lastly, 11 and 48 documents were found using the keywords “nanocellulose”, “bio-composite”, “nanocellulose”, and “biodegradable composite”, respectively. In the same way, for the environmental impact, SCOPUS was also used as the main database. The keywords “LCA”, “nanocellulose”, and “composite” were used to refine the search, and with the first two keywords, a total of 23 papers were displayed from 2015–2022. When the search was carried out with all three keywords together, only three results were revealed. The review of the life cycle focused more on the papers found in 2022, as they were not included in any other review paper published.

## 3. Processing of Nanocellulose Biobased Composites

### 3.1. Processing Techniques: Solvent Casting and Melt Processing 

Nanocellulose-based biocomposites are produced by incorporating nanocellulose (NC) reinforcement into a biopolymer matrix to enhance the overall properties of the composite. A homogeneous dispersion of the filler and appropriate compatibility between the filler and matrix is crucial to achieving the desired performance of the composite. However, good dispersion and compatibility can pose a challenge when using nanocellulose, as it tends to self-agglomerate within the polymer matrix [33,34,41]. Various processing techniques have been developed to overcome this challenge by optimizing the dispersion and interaction of the filler in biopolymer matrices. Typically, solvent casting and melt processing are two of the most commonly used methods for preparing these nanocomposites.

Solvent casting is a simple and flexible technique that utilizes a solvent to dissolve the polymer matrix at low temperatures [41,42]. This process is also referred to as solution casting when water is used as the solvent [22,26,43,44]. The nanocellulose is added to the polymer solution through mechanical stirring and ultrasonication steps to eliminate bubbles [43], producing a homogeneous composite. The film is then obtained through precipitation when the solvent is allowed to evaporate [43].

Polysaccharides are one kind of biodegradable polymer source that has drawn attention owing to their abundant supply, low cost, and biodegradability properties. Starch and chitosan have been extensively used as matrices, as seen in Table 1. Due to their hydrophilic nature, solvent casting processing can be achieved using mainly water. Louis et al. [20] prepared a biobased composite using nanocellulose extracted from rice straw and corn starch as a matrix. Ultrasonication for 5 min was performed to ensure homogenization and bubbles removal. The resulting composites showed that the addition of 1 wt.% of nanocellulose increased tensile strength from 2.96 MPa to 5.22 MPa, and reduced water vapor permeability (WVP) from 1.31 × 109 gm^−1^ s^−1^ Pa^−1^ to 0.33 × 109 gm^−1^ s^−1^ Pa^−1^. The enhancement of properties was attributed to a well-dispersed reinforcement on the polymer matrix achieved by solvent casting [20]. Moreover, Pires et al. [21] prepared cellulose nanocrystals (CNC)/chitosan composites by solvent casting at 1.5, 2.0, and 2.5 wt.% filler concentrations. The highest tensile strengths and elastic modulus were observed in composites with 2.0 wt.% load. These results were attributed to the good interaction between the cationic amine groups of chitosan with the anionic sulfate groups and hydroxyl groups in CNC. Even though 3 cycles of agitation were adopted to promote dispersion, agglomeration in the matrix was registered at 2.5 wt.% of nanocellulose content. In addition, the elongation at break decreased as increasing the filler content. This behavior was attributed to poor dispersion and orientation of the nanocellulose in the matrix. If nanocellulose were well distributed, composites would present a higher elasticity due to an efficient stress transfer between the matrix and the reinforcement [21]. Similar elongation results were obtained when higher CNC content was introduced in a chitosan matrix [45]. A decrease of 17.9% in the elongation at the break of chitosan-based composites reinforced with 10 wt.% of CNC was reported. The study indicated that the reduction in elongation was a result of the strong interaction between the reinforcement and chitosan through hydrogen bonding, which limits the mobility of the biopolymer. Although morphology was not evaluated, the absence of agglomeration in films containing 8 wt.% CNC was noted, which was attributed to the good compatibility between chitosan and CNC, both being water soluble, leading to good dispersion during the formation of the composite film.

Another study [46] reported that just above 5 wt.% of cellulose nanofibrils (CNF), the effectiveness of reinforcement is reduced since dispersion is difficult to accomplish during solvent casting processing when high fiber concentrations are added. Therefore, agglomeration is more likely to occur. On the other hand, Zeng et al. [47] described the successful incorporation of 6 wt.% of pomegranate peel CNC in a chitosan matrix using solvent casting followed by 2 h of mechanical stirring. Scanning electron microscope (SEM) images showed a homogeneous microstructure without the presence of cracks or pores, even at a high CNC concentration, which could be attributed to a well-dispersed reinforcement accomplished by an extensive period of agitation. These results agreed with Ahmad et al. [25], who also reported an even distribution of 10 wt.% of cellulose nanofibers from a banana peel in a corn starch matrix.

Some parameters of the solvent casting technique have been assessed to enhance dispersion. Temperature processing has been found to impact the distribution of nanocellulose in the matrix. Menezes et al. [48] tested two different solubilization temperatures when preparing CNF (5 wt.% load)/starch biobased composites by solvent casting. Films prepared at 70 °C exhibited the highest gain in tensile strength compared to films prepared at 90 °C due to better dispersion during the film processing. Moreover, pH was assessed during the preparation of CNF–biomass yeast composites [49]. Alkaline pH promoted solubilization, which facilitated the addition of 5 wt.% of nanocellulose in the films. A nanocellulose/yeast composite with reduced water vapor permeability, higher Young’s modulus and higher tensile strength was obtained at pH 11. Blends of polysaccharide matrices have also been produced using the solvent-casting method to improve reinforcement–matrix compatibility [50]. It has been reported that the mechanical, thermal, rheological, and permeability properties were enhanced by adding 5 wt.% of CNC to a chitosan–starch biobased matrix. However, similar to previous reports, higher concentrations of nanocellulose had a negative impact on the performance of the composite, and despite extended stirring, agglomerations could not be avoided.

Rose et al. were able to add 10 wt.% of nanocellulose fibers to an Acacia nilotica (babul gum) matrix without the formation of agglomerations [51]. The composites exhibited an increase of 93% in the elastic module compared to the pristine babul gum film. The authors claimed that the reinforcing effect obtained was mainly accomplished by the good compatibility between the babul gum and nanocellulose due to their hydrophilic nature.

**Figure 2:**
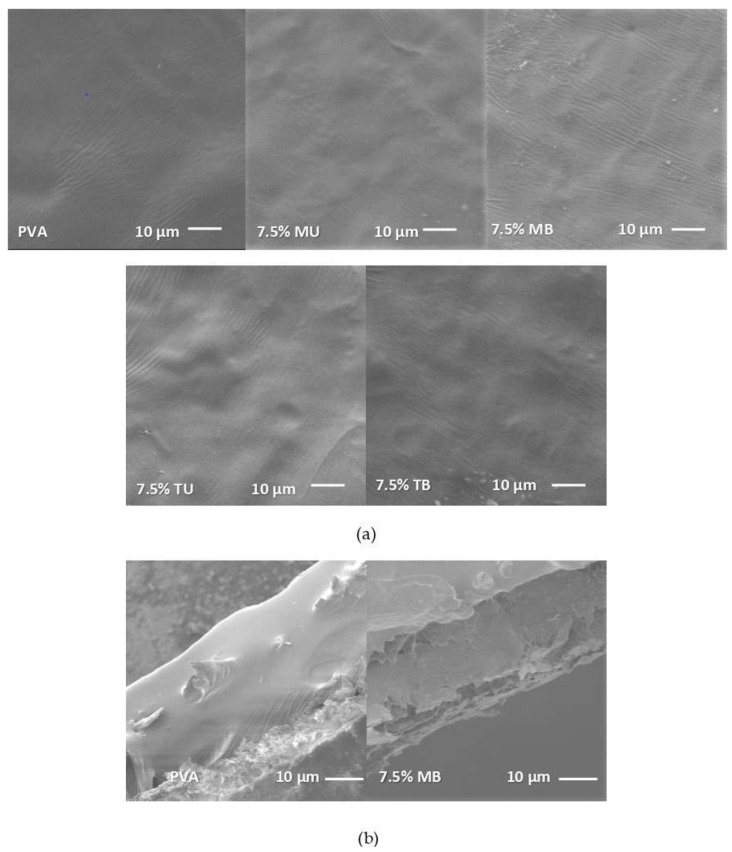
SEM micrographs of pure PVA and PVA-(L)CNF films. (**a**) the surface of pure PVA, PVA containing 7.5% of mechanical unbleached nanocellulose (7.5% MU), PVA containing 7.5% of mechanical bleached nanocellulose (7.5% MB), PVA containing 7.5% of TEMPO unbleached nanocellulose (7.5% TU), PVA containing 7.5% of TEMPO bleached nanocellulose (7.5% TB); (**b**) cross section of pure PVA and PVA containing 7.5% of mechanical bleached nanocellulose (7.5% MB). Taken from Sánchez et al. [24].

Another promising biodegradable polymer is polyvinyl alcohol (PVA). PVA is a nontoxic, water-soluble, and biodegradable synthetic polymer that, combined with nanocellulose, offers improved mechanical, thermal, and barrier properties. PVA dissolves in water, which is an advantage for composite processing, as the solvent casting of PVA biobased composites is most often done with water. [22,24]. Yudhanto et al. [22] prepared CNF–PVA composites by solution casting under constant stirring and ultrasonication process to obtain well-dispersed films. The conditions of these steps are reported in Table 1. A high concentration (8 wt.%) of nanocellulose fibers was successfully incorporated into PVA without agglomerations. This film showed excellent mechanical properties, with a substantial improvement in tensile strength (79%) and elongation at break (138%), which stands for a homogeneous stress transfer in the composite interface achieved by good adhesion and dispersion. When agglomerations are formed, there is a nonuniform distribution of the reinforcement in the matrix, which leads to the failure of the composite at lower tensile loadings [26]. For example, Srivastava et al. [52] ultrasonication for filler concentration below 5 wt.%. However, above this concentration, a drastic decrease in tensile strength and elongation at break were observed, which was a direct result of the nanocellulose self-agglomeration. Similarly, Sánchez et al. [24] evaluated the surface roughness of the CNF–PVA composites by SEM, shown in Figure 2. (provided by Sanchez et al. from Foods and Open Access Journal under the terms and conditions of the Creative Commons Attribution license). The SEM image shows that PVA films that are reinforced with a 7.5 wt.% of nanocellulose exhibited a higher surface roughness than neat PVA. This is a result of a nonhomogenous distribution of the filler, causing weak mechanical properties. It is worth noting that the previous studies mentioned [24,26,52] included stirring cycles during film preparation and short or no periods of ultrasonic steps, which have not helped dispersion when adding high reinforcement loads (>5 wt.%). Following this approach, Somvanshi and Gope [43] suggested that the agglomeration of nanocellulose occurs when the solvent is evaporated. Therefore, a modified solvent casting method involving sonication was proposed to improve the dispersion of nanocellulose in a polyvinyl alcohol (PVA) matrix. Films produced using this method, which included 30 min of ultrasonication after solvent casting, showed superior mechanical properties compared to composites produced without sonication. In particular, the elastic modulus was found to be 45% higher for 40 wt.% CNF–PVA films prepared with sonication compared to films without sonication. The results suggested that good dispersion of nanocellulose in PVA achieved during sonication promotes intermolecular interaction, and then stronger composites are formed. Moreover, the higher thermal stability of ultrasonicated films with 40 wt.% of CNF was obtained (12.8 °C superior to films without sonication) due to the increased crystallinity arrangement of nanocellulose promoted by the sonication step.

Ultrasonication has also been successfully implemented in the preparation of polylactic acid (PLA)-based nanocomposite [23]. PLA has drawn attention due to its biodegradability and biocompatibility as it is synthesized from renewable agricultural raw materials [33]. In addition, PLA is a hydrophobic polymer, so solvent casting is usually achieved by other solvents than water. Zhang et al. [23] reinforced PLA with CNC obtained from coconut shells by solvent casting. Ultrasonication followed solvent casting to uniformly disperse the nanocellulose in the biopolymer (see processing conditions in Table 1). SEM images of PLA films reinforced with 3 wt.% of CNC showed a typical sea-island structure, which demonstrates an evenly disperse system. Nevertheless, higher concentrations of reinforcement (4 wt.% and 5 wt.%) decreased impact strength, flexural strength, and modulus due to the formation of agglomerates in the PLA.

Solvent casting is an effective processing technique for incorporating nanocellulose in various biopolymer matrices. Achieving a well-dispersed composite without the formation of clumps is crucial for obtaining improved mechanical, thermal, and permeability properties compared to the original polymer films. To this end, various methods have been explored to promote uniform dispersion, such as mechanical stirring, ultrasonication, temperature control, and pH regulation. However, studies report problems when adding higher nanocellulose content (>5 wt.%), even with the incorporation of the above-described solvent casting modifications. Niinivaara et al. [53] explained that the reinforcement effect depends not only on the dispersion of filler in the matrix but also on the fiber orientation, which is affected by drying and speed of processing. In this sense, a recent study reported that an even orientation and distribution could be bolstered by producing granule-size composites by melt processing and then reshape them by compression molding [54].

**Table 1:** Solvent casting processing conditions of nanocellulose biobased composites.

Reinforcement (Source)	Matrix	Solvent Casting Conditions	Mechanical Properties		
			Tensile Strength (MPa)	Young’s Modulus (MPa)	Elongation (%)
**Polysaccharide matrix**					
**NC** **(rice straw)** [20]	Corn starch	Solvent: water NC content: 0.25–1 wt.% Plasticizer: 25 wt.% glycerol Temperature: 85 °C Sonication for 5 min	3.0–5.2	82.3–200.8	19.4–45.4
CNC (biomass) [21]	Chitosan	Solvent: 1 % *v*/*v* glacial acetic solution NC content: 0, 1.5, 2.0, 2.5 wt.% Plasticizer: 30 wt.% glycerol Agitation for 5 min at 15.000 rpm Sonication for 15 min	34.0–43.8	1415.4–2221.8	10.9–27.9
CNF (Orange bagasse) [48]	Starch	Solvent: deionized water NC content: 5 wt.% Plasticizers: glycerol Temperature: 70 °C and 90 °C Agitation for 2 h at 1200 rpm Ultrasonication at 42 kHz for 10 min	-	-	-
CNC (pea pod) [45]	Chitosan	Solvent: 2 wt.% acetic acid solution. NC content: 0–10 wt.% Mechanical stirring for 15 min at room temperature	52.2–73.4	880.0–1692.0	11.2–17.9
BC and CNF (rice husk) [49]	Yeast Biomass	Solvent: water. NC content: 0, 1, 3, and 5 wt.% Plasticizer: 25 wt.% glycerol pH: 6 and 11 Mechanical stirring for 1 min Homogenization at 125 MPa for 9 min.	1.0–3.5	20.0–36.0	11.0–44.0
CNC (pomegranate peel) [47]	Chitosan	Solvent: 1% *v*/*v* acetic acid glacial. NC content: 0–6 wt.% Plasticizer: 30 wt.% glycerol Mechanical stirring for 2 h at room temperature Ultrasonication for 5 min	28.9–41.3	59.0–96.7	88.9–95.8
CNF (açaí) [46]	Chitosan	Solvent: 0.5% *v*/*v* acetic acid solution. NC content: 5–20 wt.% Plasticizer: 30 wt.% glycerol Mechanical stirring for 30 min at room temperature	6.5–9.7	458.6–1119.9	-
CNC [50]	Chitosan-corn starch	Solvent: 1% *w*/*v* in acetic acid solution. NC content: 0–10 wt.% Plasticizer: 30 wt.% glycerol Mechanical stirring for 10 min at 90 °C.	2.9–13.6	-	5.3–145.1
CNF (Bamboo fibers) [51]	Babul gum	Solvent: distilled water. NC content: 0–10 wt.% Plasticizer: 10 wt.% sorbitol Mechanical stirring for 30 min at 85 °C.	0.4–3.7	4.8–71.7	17.5–46.0
CNF (banana peel) [25]	Corn starch	Solvent: distilled water. NC content: 0–15 wt.% Plasticizer: 25 wt.% glycerol Mechanical stirring for 30 min at 81 °C Sonication for 10 min	7.0–9.0	-	20.0–25.0
**PVA matrix**					
CNF (agave) [22]	PVA	Solvent: distilled water NC content: 0–10 wt.% Mechanical stirring for 30 min at 50 °C and 350 rpm. Ultrasonication for 2 min	26.6–47.0	-	33.0–112.0
NC [52]	PVA-banana pseudo fiber	Solvent: distilled water NC content: 0–5 wt.% Plasticizer: 10 wt.% sorbitol Mechanical stirring for 40 min Ultrasonication	28.7–43.8	-	87.4–183.0
CNF (sugar cane bagasse) [43]	PVA	Solvent: distilled water NC content: 0–50 wt.% Plasticizer: 70 wt.% PEG Mechanical stirring for 60 min Ultrasonication for 30 min	26.5–85.4	954.0–2846.0	7.8–91.1
CNF (olive tree pruning) [24]	PVA	Solvent: distilled water NC content: 0, 2.5, 5.0, and 7.5 wt.% Mechanical stirring for 4 h at room temperature.	52.5–69.8	3587.0–4263.0	100.0–143.0
CNC (sugar cane bagasse and coir fiber) [26]	PVA	Solvent: distilled water NC Content: 0.5 and 2.0 wt.% Mixing Sonication for 5 min	19.4–38.2	11.6–26.4	105.8–187.0
CNF (Coconut shell) [44]	PVA	Solvent: distilled water NC content: 2 wt.% Mixing at a warm temperature	2.6–6.7	-	36.2–102.44
**PLA matrix**					
CNC (office wastepaper) [23]	PLA	Solvent: N, N-dimethyl formamide solution NC content: 1–5 wt.% Mechanical stirring for 4 h at 60 °C. Ultrasonication	61.0–69.0	-	5.0–6.0

Melt processing is another commonly utilized method to prepare composites, in which reinforcement and matrix are mixed and melted in extruders or melt mixers [55,56,57] without the need for additional solvents. Table 2 shows the control parameters such as temperature, pressure, rotating speed, and residence time for various nanocellulose biobased composites prepared by melt-processing techniques. Melt processing is a versatile technique that offers various advantages. First, polymer blend composites can be effectively fabricated by melt processing techniques to add some valuable properties to composites [58]. In a recent study [59], PLA was blended with poly (butylene succinate) (PBS) to enhance melt processability, thermal stability, and mechanical properties and to reduce costs. Atomic force microscopy (AFM) results of CNC–PLA–PBS blend composites confirmed good miscibility between PBS and PLA and good dispersion of CNC into the blended matrix at different nanocellulose loadings. Moreover, SEM images revealed that there was no debonding when composites were fractured, suggesting good interaction between fibers and matrix. The authors concluded that the fabrication method used to prepare this blend composite resulted in a well-dispersed filler within the polymer matrix, which contributed to improved tensile strength and elongation at break when the matrix contained up to 1 wt.% of CNC. Another benefit of melt processing is that the dispersion of the fibers is enhanced by the shear forces generated by extruders and melt mixers. [56]. Copenhaver et al. [56] reported a reinforcing effect when adding high loads of CNF (10–40 wt.%) to PLA by melt compounding. An increase of 73% in Young’s modulus was seen for composites with 40 wt.% of nanocellulose fibers. According to the authors, increasing the shear forces during processing could improve the dispersion of the fibers, for shear forces disrupt hydrogen bonding between fibers, and then agglomeration is prevented. Nevertheless, increasing shear forces and/or residence time could degrade the biopolymer and increase energy consumption. Another alternative to reduce agglomeration has been presented by Chihaoui et al. [60]. This study found that using polyethylene glycol (PEG) as a carrier during the melt processing of lignin-containing cellulose fibrils (LCNF)–polylactic acid (PLA) composites reduces the tendency of the fibers to self-aggregate. The LCNF, PLA, and PEG create an interconnected network. Adding 8 wt.% LCNF to the matrix resulted in a 243% increase in tensile strength and an 1100% increase in Young’s modulus. These excellent results were not only attributed to the addition of PEG but also to the improved interaction between the fibers and the matrix due to the use of LCNF.

PLA is an excellent material for melt processing due to its low melting temperature (~180 °C) [60]. On the other hand, other biopolymers, such as PVA, do not exhibit good melt processability. In the case of PVA, its melting temperature is close to the degradation temperature due to the high crystallinity and strong intra- and inter-hydrogen bonding, which makes melt processing difficult. Zhou et al. [61] reported a method to increase the melt processing window and reinforcement effect by adding CNF and corn starch to a PVA matrix. Initially, PVA and reinforcement were dissolved in water and formamide to destroy the regularity of PVA polymer chains, reducing crystallinity and thus decreasing melting temperature. In addition, disrupting the regular structure of PVA produced a greater interaction between the PVA and the hydroxyl groups of the nanocellulose, so a higher temperature of degradation was seen, which finally increased the melt-processability of PVA. The addition of 10 wt.% of CNF and 20 wt.% of corn starch increased the melt processing window from 92.14 °C to 132.99 °C. Furthermore, tensile testing revealed that the reinforcement effect was improved. The tensile strength increased by 25% when PVA was reinforced with 10 wt.% CNF and 10 wt.% corn starch. This new melt processing approach enhanced dispersion and interaction between the matrix and filler when higher filler concentrations were added. Despite the fact that agglomerations appeared in 20 wt.% CNF–PVA composites, higher nanocellulose loadings have been successfully introduced compared with conventional solvent casting methods.

### 3.2. Effect of Plasticizers and Additives

Whether nanocellulose biobased composites are prepared by solvent casting or melt processing, plasticizers are part of the composite composition, for they favor dispersion and enhance the flexibility, barrier properties [21], and processability [61] of the composites. Some of the most common plasticizers used are glycerol and sorbitol (see Table 1 and Table 2) due to their high compatibility with biodegradable polymers.

Pati et al. [63] researched the effect of nanocellulose (0.26–0.95 *w*/*v*.%), glycerol (1.16–2.84 *w*/*v*.%), and PVA (0.66–2.34 *w*/*v*.%) concentrations on the tensile properties of a starch film reinforced with nanocellulose. Although the nanocellulose load caused the major gain in mechanical properties, films with high plasticizer concentrations and PVA exhibited high tensile strength. However, researchers reported that after a certain glycerol concentration, tensile strength decreased due to the plasticizing effect that weakened the starch–nanocellulose interface. On the other hand, PVA helped with the gain in tensile strength since its structure contains more hydroxyl groups. Moreover, the results showed that combining high concentrations of glycerol and PVA promoted chain mobility and sliding, favoring composite flexibility. The authors concluded that the optimum composite with improved functionality was the composite with 0.89% NC, 2.53% glycerol, and 1.89% PVA due to the high tensile strength (8.92 MPa), elongation at break (41.92%), and reduced water vapor permeability (7.07 × 10^−10^ g m^−1^ s^−1^ Pa^−1^). The impact of the plasticizer type (sorbitol and glycerol) and concentration (30% and 40%) in the properties of a cellulose nanocrystal–thermoplastic starch (TPS) composite has been reported by Csiszár et al. [64]. The authors explained that the reduced strength observed at high plasticizer concentrations was due to the competitive interaction between the starch, nanocellulose, and plasticizer, which impeded the interaction between the matrix and the reinforcement. Additionally, the effectiveness of the plasticizer depended on its compatibility with the biopolymer. In this study, glycerol was found to have higher compatibility with starch, leading to lower strength and higher flexibility than sorbitol. Nazrin et al. [54] reported that sorbitol and glycerol have a plasticizing effect and modify the composites’ flammability. Although both are flammable substances, glycerol has a higher potential to increase the flammability of CNC–polylactic acid thermoplastic starch (PLA–TPS) composites than sorbitol due to its lower molecular weight and ability to migrate into the biopolymer matrix.

Besides plasticizers, other additives have been tested to improve the functionality of nanocellulose biobased composites. Santos et al. [65] reported that the addition of 2 wt.% of papain, a proteolytic enzyme, in nanocellulose–chitosan composites produced a more thermally stable film by increasing the initial degradation temperature by 9.7 °C. Essential oils have also been incorporated into composites to improve the hydrophobicity, antioxidant, and antimicrobial properties of a CNF–PVA composite [44]. Contact angle tests indicated that the addition of linseed (0.5%) and lemon (0.5%) oils improved the surface hydrophobicity of the composite. The contact angle increased from 53° to 91°, suggesting a nonwettable film surface. Moreover, reduced water absorption and improved antimicrobial and antioxidant properties were obtained, which are ideal for food packaging applications. These results are consistent with the ones reported by Bangar et al. [66]. The incorporation of clove bud oil into a CNC–starch composite not only reduced water vapor permeability from 9.6 × 10^10^ to 7.25 × 10^10^ g m^−1^ s^−1^ Pa^−1^, it also increased film flexibility and tensile strength by 8% and 14%, respectively. The addition of essential oil may have a plasticizing effect. Another additive with the antimicrobial effect that has been included in nanocellulose composite formulation is poly hexamethylene biguanide (PHMB) due to its broad antibacterial spectrum and high compatibility with biopolymers [67]. It was reported that the addition of PHMB to nanocellulose–starch composites enhanced the films’ antimicrobial properties, flexibility, and thermal stability. Furthermore, Zhao et al. [68] reported that the addition of lignin to a CNF–starch based composite improved the hydrophobicity and UV-blocking properties of these composites. These enhancements were attributed to the hydrophobic nature and unique structure of lignin.

## 4. Effect of Nanocellulose Content in the Morphology and Properties of Biobased Composites 

### 4.1. Morphological and Structural Properties

Morphology characterization of nanocellulose biobased composites is mainly done using scanning electron microscopy (SEM) and atomic force microscopy (AFM).

The SEM surface images of starch films reinforced with nanocellulose were reported by Louis et al. [20]. Nanocellulose content at 0.75 wt.% increased roughness at a nanoscale level, however, the uniformity and smoothness of the films were not altered. Zeng et al. [47] assessed the surface and cross-sectional morphology of chitosan biocomposites reinforced with different crystalline nanocellulose loads prepared by solvent casting. There was no evidence of cracks, pores, or agglomerations up to 6 wt.% CNC loads due to the homogeneous dispersion of nanocellulose inside the matrix. However, higher reinforcement concentrations have shown the formation of nonhomogeneous composites. Yudhanto et al. [22] and Oyeoka et al. [69] found that nanocellulose formed agglomerations when their concentration was above 8 wt.% concerning the PVA matrix. High reinforcement loadings have been shown to decrease dispersion due to the high amount of filler in the matrix, which leads to the aggregation of nanocellulose [22,50,69]. Moreover, SEM images of fractured composites have revealed important information about the interaction between nanocellulose and PLA during tensile fracture [55]. Homogeneous distribution of 3 wt.% of CNF fibers was observed in the fractured composites. On the other hand, the presence of large clusters of nanocellulose was evidenced in composites with 6 wt.% of nanocellulose. Biobased composites with three wt.% of CNF presented a higher reinforcing effect than composites with 6 wt.% due to a stronger interaction between the nanocellulose and biopolymer. Similar findings were also found in the SEM micrographs of 3 wt.% and 7 wt.% CNC–PVA films [70]. Composites with higher CNC content exhibited lower mechanical properties due to the presence of voids and agglomerates in the fracture surfaces, resulting in weak stress transfer through the interface. Further, Rasheed et al. [59] conducted an AFM analysis to investigate the morphology and miscibility of bamboo nanocellulose in the PLA–PBS matrix prepared by melt processing. Results showed an evident increase in the surface roughness and dispersion with the addition of CNC from 0–1.5 wt.%. However, SEM analysis of fractured composites demonstrated the presence of voids and agglomerations in composites with 1.5 wt.% of CNC, reducing the film’s tensile properties.

Structural properties are mainly determined by X-ray diffraction (XRD) and Fourier transform infrared spectroscopy (FITR), which are used to assess the biocomposites’ crystallinity and chemical composition, respectively.

Pires et al. [21] reported that adding small amounts of nanocellulose (2.5 wt.%) to a chitosan matrix did not extensively change the FTIR spectra of chitosan due to the similar chemical structures of both components that form a strong interaction. Similar results were reported by Hamid et al. [45] who investigated the chemical structure of chitosan films reinforced with CNC by FTIR. The increment of nanocellulose from 1–10 wt.% caused slight shifts in the peaks at 1552 cm^−1^, 1643 cm^−1^, and 3200 cm^−1^, corresponding to NH-bending (amide II), NHCO (amide I), and NH and OH-stretching vibrations. These changes were attributed to the interaction between the hydroxyl groups of crystalline nanocellulose and the amine groups of chitosan, owing to forming a strong interface through hydrogen bonding [47]. Similar results were highlighted by Kalajahi et al. [71], who reported the preparation of nanocellulose biobased composites using orange waste powder (OWP) as a matrix. FTIR spectra showed that the addition of 6 wt.% of nanocellulose caused a shift in the OH-stretching peaks due to hydrogen bond formation between the matrix and the fillers.

The XRD patterns of chitosan films reinforced with nanocellulose were reported by Pires et al. [21]. The peaks in the X-ray diffraction at 11.4° and 22.5° confirmed the presence of both crystalline and amorphous structures of the chitosan, respectively. The addition of 2.5 wt.% of CNC slightly increased the intensity of the peak at 22.5°, which is related to the crystalline cellulose, suggesting the successful addition of nanoparticles in the biopolymer. Finally, a higher crystalline index was achieved for chitosan films reinforced with nanocellulose. Similar results were reported by Yudhanto et al. [22] for PVA reinforced with agave cellulose nanofibers composites. The addition of different reinforcement loads did not change the peak position (2θ = 22.84°), however, a gradual increase in the peak intensity was observed, confirming the addition of nanocellulose. Additionally, the incorporation of 8 wt.% of CNF led to a 22% increase in crystallinity compared to a pristine PVA film. Several studies have confirmed that adding nanocellulose to the biopolymer phase enhances crystallinity due to introducing a crystalline phase (nanocellulose) and forming uniform films [50,71]. According to Chou et al. [72], adding nanocellulose also results in a larger crystal grain size in CNC–PVA composites.

### 4.2. Mechanical Properties

The reinforcing effect of nanocellulose in the different biopolymer matrices is commonly assessed by mechanical properties such as tensile strength, Young’s modulus, and elongation at break. Tensile testing of biocomposites is usually carried out using standard methods, as per ASTM D882 [73] and ASTM D638 standards [20,24,26,55].

Hamid et al. [45] studied the reinforcing effect of nanocellulose derived from pea pod on chitosan as a function of the nanocellulose loading. The tensile strength and Young’s modulus increased as the reinforcing concentration increased from 1 to 10 wt.%. Composites with 10 wt.% of nanocellulose increased in tensile strength from 52.2 MPa (neat chitosan) to 73.4 MPa, and Young’s modulus from 880 MPa (neat chitosan) to 1692 MPa. Similar results have been reported in a recent study in which the mechanical properties of CNF–starch composites prepared with different filler loadings were evaluated [62]. Biocomposites with 5 wt.% of nanocellulose increased by 67% and 134% in tensile strength and Young’s modulus compared to the thermoplastic starch, respectively. Further increase in the reinforcement load did not enhance the mechanical properties, which was attributed to the self-aggregation of nanocellulose in the matrix. When nanocellulose is added to the biopolymer, if there is a good dispersion, an intermolecular network is formed through hydrogen bonding. Therefore, the addition of higher concentrations of nanocellulose increases the availability of hydroxyl groups for hydrogen bonding, which permits the formation of a stronger interface [21]. Nevertheless, if the reinforcement material is not well dispersed through the matrix and forms aggregates, then mechanical properties are diminished because agglomerates cannot bear loads due to lower matrix-filler interactions [52,74]. This effect was confirmed by Shazleen et al. [55]. The tensile strength and Young’s modulus of CNF–PLA composites, prepared through melt processing, increased with the incorporation of up to 4 wt.% of nanocellulose. However, at higher concentrations, an adverse effect on the tensile strength and Young’s modulus was observed, as confirmed by the formation of aggregates by scanning electron microscopy (SEM) images. Moreover, the increase in stiffness was attributed not only to the addition of rigid nanoparticles that restrict the mobility of PLA chains and also to the crystalline nature of CNF, which functions as a crosslinking agent. On the other hand, the enhancement of tensile strength was attributed to the hydrogen bonding between the matrix and filler. Pavalaydon et al. [26] reported that PVA composites prepared with nanocellulose from different sources exhibited different mechanical properties. Higher tensile strength was observed for composite prepared with 0.5 wt.% (38.2 MPa) and 2 wt.% (32.8 MPa) of nanocellulose from coir fiber than those prepared with 0.5 wt.% (23.4 MPa) and 2 wt.% (24.9 MPa) of nanocellulose from sugarcane bagasse. Infrared spectra showed that coir fibers exhibited a greater band intensity corresponding to hydroxyl groups (3300 cm^−1^ and 1029 cm^−1^), which ultimately represented higher hydrogen bonding with the matrix and, thus, higher mechanical properties.

Zeng et al. [47] also reported the mechanical properties of chitosan composites reinforced with different nanocellulose loads (0–6 wt.%) obtained from pomegranate peel. Even though the tensile strength increased with the addition of filler, the gain in tensile strength was lower than the study presented by Hamid et al. [45], who also used chitosan as a matrix. The authors explained that the enhancement of tensile properties depends not only on the type of nanocellulose, filler load, and its dispersion, it also depends on the aspect ratio (length/diameter) of the nanocellulose, which also plays an important role. Higher aspect ratios of nanocellulose have been shown to improve tensile strength and Young’s modulus, for stress transfer is more uniformly distributed through the interface when the composite is under tensile loads [21]. For example, chitosan reinforced with 5 wt.% of nanocellulose derived from a pea pod with an aspect ratio of 89 exhibited a tensile strength of around 65 MPa, compared with chitosan films reinforced with 6 wt.% of nanocellulose with an aspect ratio of 25 that showed a tensile strength of about 40 MPa [45,47].

The elongation at break reflects the flexibility of the biobased composites, and it is affected by the biopolymer chain mobility [47,51]. Hamid et al. [45] reported that the elongation at break showed an inverse relationship with the nanocellulose concentration due to the reduced mobility of the biopolymer chains. At 10 wt.% of the filler loads, the nanocellulose–chitosan composite exhibited an elongation at a break of 37% lower than the chitosan film. A similar trend was observed by Pires et al. [21], who reported the reduction of elongation at break in 75% of chitosan films with the addition of 2.5 wt.% of crystalline nanocellulose. The decrease in the composite flexibility could be explained by the reduced mobility due to the presence of rigid nanoparticles [69]. The authors explained that such behavior owes to a lack of orientation and dispersion of the filler in the matrix. A study by Yudhanto et al. [22] revealed that the elongation at the break of PVA increased with the addition of CNF. The addition of 8 wt.% of CNF increased the elongation from 47% to 112%. This behavior shows a well-distributed nanocellulose in the matrix, causing an effective stress transfer of the nanocomposite. Several stirring and ultrasonication steps were performed to achieve homogeneous distribution during the composite preparation by solvent casting. Zhang et al. [23] reported similar results in their study of the mechanical properties of PLA films reinforced with crystalline nanocellulose at various concentrations (1–5 wt.%). Composites with 4 wt.% displayed the highest tensile strength and elongation at break, which increased by 9.7% and 5.8%, respectively. Furthermore, the impact and flexural strength were assessed. A proportional relationship between these properties and the filler concentration was observed for composites up to 3 wt.%. Higher concentrations of reinforcement resulted in a reduced ability to deform due to the strong interactions between the nanofillers and the matrix [58].

### 4.3. Thermal Behavior

Differential scanning calorimetry (DSC) is used to determine the thermal parameters of composites, such as glass transition point (T_g_), melting temperature (T_m_), heat capacity (ΔH), and degree of crystallinity (X_c_).

Yudhanto et al. [22] reported that the addition of 2–10 wt.% of CNF on a PVA matrix increased T_g_ and T_m_ due to the mobility restriction generated by intermolecular interaction between the filler and matrix and the addition of crystalline domains in the polymer matrix, respectively. In addition, X_c_ increased from 22.2% to 43.5% when 8 wt.% of nanocellulose was added. These results agree with various studies [55,71,75,76,77] that have reported that the inclusion of nanocellulose increased the composite crystallinity since the nanoparticles act as nucleation agents, thus facilitating crystallization. Shazleen et al. [55] studied the crystallization kinetics using a DSC analysis of CNF–PLA composites reinforced with 1–6 wt.% of nanocellulose. Results revealed that the addition of up to 3 wt.% of CNF increased the degree of crystallinity from 2.3% to 44.2% and increased the crystallization rate from 0.011 min^−1^ to 0.716 min^−1^ during isothermal crystallization (T_c_ = 100 °C). The nucleation effect of nanocellulose was enhanced due to its good distribution in the PLA matrix. However, a reduced crystallinity degree was obtained for composites with 4 wt.% to 6 wt.% due to the agglomeration of nanocellulose, which diminished crystallization. The enhancement in crystallization caused by the addition of nanocellulose also contributed to the improvement of mechanical properties. Contradictory results were reported by Zhou et al. [61], who presented the DSC curves of PVA films reinforced with different ratios of corn starch and CNF. Two peaks were observed in the DSC curves at 160 °C and 230 °C, which were attributed to the melting and degradation processes of PVA, respectively. The melting peak shifted to lower temperatures as the ratio of starch/CNF increased, for the fillers restrained the crystallization of PVA, thus decreasing T_m_. These results were confirmed by the X_c_ and ΔH values calculated from the DSC analysis, which also showed the same decreasing trend of T_m_. Furthermore, Ahmad et al. [25] reported the DSC thermograms of different CNF–starch composites, where the effect of the nanocellulose concentration in the composite degradation was assessed. All the DSC curves presented a unique broad endothermic peak, with a peak temperature that increased nanocellulose from 106.22 °C to 112.66 °C when 5 wt.% and 15 wt.% of CNF were added to the starch matrix, respectively.

Thermal degradation of biobased composites is assessed by thermogravimetric analysis (TGA) and the derivative thermogravimetric analysis (DTG). Temperatures that are assessed correspond to T_onset_ which is the temperature of the initial degradation and T_max_, which is the temperature at which the maximum degradation is registered.

A study of the thermal degradation of chitosan reinforced with 1–10 wt.% of nanocellulose revealed that the degradation behavior of these composites occurred in two stages [45]. In the range of 80–120 °C, the water adsorbed by the composite was evaporated. Then, the major weight loss occurred between 220 °C and 360 °C, where chitosan’s polymer chains were depolymerized, and glycosidic bonds of cellulose were cleaved. Furthermore, the addition of nanocellulose increased the T_max_ due to increased interaction between the matrix and the reinforcement. A similar trend was observed by Yudhanto et al. [22], who reported that the thermal stability of a PVA film was enhanced by increasing the filler concentration from 2–8 wt.% of CNF. Above this concentration, a slight decrease in T_max_ was observed, likely due to the weakened interaction between the matrix and nanocellulose. Oyeoka et al. [69] reported similar findings in their study of the thermal properties of CNC/PVA composites. The DTG plots revealed that adding 10 wt.% of crystalline nanocellulose increased T_onset_ and T_max_ from 204–209 °C and 380–385 °C, respectively. The authors suggested that the increased thermal stability obtained was due to a barrier effect created by the presence of nanocellulose particles that impede the volatilization of byproducts produced during degradation. Other authors [51] attributed the improvement in thermal stability in nanocellulose biobased composites to the crystalline nature and chemical structure of the reinforcement.

### 4.4. Thermomechanical and Rheology Analysis 

Dynamic mechanical analysis (DMA) is used to determine the temperature dependence of the tensile storage modulus (E’) and loss tangent (tanδ) of the nanocellulose-based composites. Oyeoka et al. [69] evaluated the thermomechanical properties of neat PVA–gelatin film, and 5 wt.% and 10 wt.% CNC/PVA–gelatin composites in the temperature range of 30–200 °C. The storage modulus curves of all films tested significantly decreased with increasing temperature. However, neat PVA–gelatin film exhibited a steeper decrease in the storage modulus than films reinforced with CNC. Nanocomposites reinforced with 5 wt.% of CNC showed higher E’ in all the temperature ranges. When the temperature increases, polymer chains gain mobility and begin to loosen up, increasing free volume and decreasing the storage modulus [58]. Therefore, the less steep reduction in the storage modulus of the CNC/PVA–gelatin matrix is due to the formation of an interconnected network that limits polymer mobility. This effect was also confirmed with the results of T_g_ obtained in the tanδ curves. The glassy transition temperature increased from 78 °C to 98 °C, with the addition of 5 wt.% of nanocellulose. This result showed a strong interaction and compatibility between the reinforcement and the polymer matrix.

The rheological behavior of nanocomposites as a function of the nanocellulose concentration has also been reported [50,62]. Kesari et al. [62] assessed the melt flow index (MFI) of 1–5 wt.% CNF–thermoplastic starch (TPS) composites according to the ASTM D1238-10 standard [72]. The MFI results were 2.08 g/10 min, 1.84 g/10 min, 1.51 g/10 min, and 1.40 g/10 min for neat TPS, 1 wt.%, 3 wt.%, and 5 wt.% nanocomposites, respectively. As expected, while increasing the reinforcement concentration, the melt flow index decreased, which means that viscosity increased. The addition of rigid nanoparticles and their strong interaction with the matrix through hydrogen bonding reduces the nanocomposite’s capacity to flow. The authors also attributed these results to the presence of self-agglomerated nanocellulose, which also reduces the mobility of the molecules. Moreover, Díaz-Cruz et al. [50] studied the rheological behavior of CNC/starch–chitosan composites by determining the apparent viscosity as a function of the nanocellulose load. The results confirmed the strong inverse relationship between the apparent viscosity and the shear rate at all the concentrations tested, which is a result of the disentangling of polymer chains. At a constant shear rate, increasing the crystalline nanocellulose concentration up to 5 wt.% increased viscosity due to the flow restrictions caused by the presence of the nanoparticles.

### 4.5. Barrier and Wettability Properties

Nanocellulose has proved to enhance not only the mechanical and thermal properties of biopolymers but also barrier properties, making nanocellulose based-composites appropriate for food packaging applications.

Water vapor permeability (WVP) reflects the amount of vapor water that permeates the composite per unit of area and time. Low WVP is desirable as it ensures that food will keep its quality under handling and storage conditions. It has been reported that the presence of crystalline nanocellulose restricts the water vapor to permeate the composite. Consequently, increasing the nanocellulose content, the WVP decreases if the reinforcement is well dispersed and evenly distributed in the matrix [21,63]. For example, Ahamad et al. [25] showed that starch films reinforced with 10 wt.% of cellulose nanofibers from banana peels reduced WVP by 36%. Comparable results were reported by Zeng et al. [47] who incorporated crystalline nanocellulose from pomegranate peels into chitosan films by solvent casting. The addition of 6 wt.% of CNC reduced the WVP by 20%, from 1.64 × 10^10^ g m^−1^ s^−1^ Pa^−1^ to 1.32 × 10^10^ g m^−1^ s^−1^ Pa^−1^. The formation of hydrogen bonds and the tortuous path formed by the presence of CNC were, in the main, responsible for the reduced diffusion of water vapor through the composite. Furthermore, Chou et al. [73] compared the WVP of PVA films reinforced with CNC and CNF. Lower WVP values were obtained for composites reinforced with CNF than those reinforced with CNC at the two filler concentrations tested (0.5 wt.% and 1 wt.%). This indicated that nanocellulose fibers formed a denser entanglement with the matrix, resulting in a more efficient network to prevent water vapor permeation.

Water vapor transmission rate (WVTR) is another parameter that is measured to assess the permeability of water vapor. Kesari et al. [62] evaluated the WVTR of different CNF–thermoplastic starch (TPS) composites according to the ASTM E96-95 standard [78]. Results indicated that even though 5 wt.% CNF–TPS composites presented some agglomeration, the WVTR was reduced to 11.60 g m^−2^ day^−1^ from 15.49 g m^−2^ day^−1^ (neat TPS) owing to the dense network formed through hydrogen bonding. Moreover, Sánchez-Gutierrez et al. [24] assessed the oxygen transmission rate (OTR) of PVA films reinforced with CNF from olive tree pruning. The OTR measures oxygen permeability, which is a fundamental parameter for food packaging applications, as low oxygen permeability is desirable to extend the food shelf-life. While pure PVA exhibited an OTR of 3.75 cc m^−2^. 24 h, the incorporation of 5 wt.% of CNF drastically reduced the OTR of the composites by 83–99%. The authors reported that the presence of lignin in nanocellulose allowed for the building of a denser network, creating an even higher oxygen barrier. Composites prepared with ligno-CNF exhibited an OTR of 0.08 cc m^−2^. 24 h compared to 0.64 cc m^−2^. 24 h of CNF without lignin. Furthermore, George et al. [74] assessed both the WVTR and OTR of CNC/PVA–starch composites prepared by solution casting. Both parameters decreased with the addition of 3 wt.% of nanocellulose. Composites with 3 wt.% of CNC exhibited a WVTR and OTR of 17.25 g m^−2^ day^−1^ and 93.65 cc m^−2^. 24 h compared to 122.47 g m^−2^ day^−1^ and 277.63 cc m^−2^. 24 h, respectively. The reduction in these parameters indicates that these films exhibited promising properties for food packaging applications. Finally, another parameter that has been reported to have a significant effect on the reduction of WVP and OP is the cellulose aspect ratio. Pires et al. [21] assessed the barrier properties of chitosan films reinforced with micro and nanocellulose of different aspect ratios. Results revealed that composites reinforced with fillers of a higher aspect ratio promoted crystallization, so a more tortuous path for vapor water and oxygen was formed. Therefore, WVP and OP were reduced.

Several studies [21,47,62] have investigated the wettability properties of biocomposites to evaluate their interaction with aqueous food matrices. Pires et al. [21] reported the swelling and solubility of different CNC/chitosan composites. The results showed that adding 2.5 wt.% of CNC reduced the swelling and solubility percentages from 174.9% and 23% to 130.1% and 17.6%, respectively, compared to neat chitosan. These findings confirmed the formation of hydrogen bonds between the filler and matrix, which reduced the availability of hydroxyl groups and free volume within the material. Zeng et al. [47] suggested that the reduction in water solubility could also be attributed to the hydrophobic barrier formed by the presence of crystalline cellulose. Moreover, Pires et al. [21] measured the contact angle of CNC–chitosan composites. The contact angle increased from 78° (pristine chitosan) to 89° (2.5 wt.% CNC). The contact angle increased by increasing the nanocellulose content, and the films became more hydrophobic. This showed that OH groups interacting with water molecules were forming hydrogen bonds. Therefore, wettability properties were reduced. This increase in hydrophobicity measured by the contact angle was also reported in other studies [51,62]. Kesari et al. [62] suggested that the improvement of contact angle could also be attributed to the increased roughness of the films caused by the incorporation of nanocellulose particles in the biopolymer matrix.

Ahmad et al. [25] reported that adding 5 wt.% and 10 wt.% of cellulose nanofibers to a starch matrix caused a reduction in the water solubility of 7% and 13%, respectively. These results showed that the water diffusion through the matrix became difficult due to the formation of a network between the filler and the matrix. However, nanocomposites prepared with 15 wt.% of reinforcement exhibited a slight increase in water solubility compared to neat starch. The authors suggested that the natural hydrophilicity of starch could play an essential role in wettability properties. Furthermore, water uptake was assessed at 25 °C, 95% relative humidity, and over 200 h. Two regions were observed. In the first region, from 0 to 100 h, water absorption increased at a faster rate. Above 100 h, the water absorption rate slowed, and a plateau zone formed. The stability of the water absorption was attributed to the strong interaction through hydrogen bonding between nanocellulose and starch that decreased the diffusivity of water within the network. It is worth noting that CNF–starch composites exhibited lower water uptake than neat starch during the whole period.

Oyeoka et al. [69] reported the water absorption of CNC–PVA composites submerged in both water and saline water for 130 min. PVA is a highly hydrophilic material. The results showed that a neat PVA film started to degrade after 30 min of exposure to water. However, the addition of 5 wt.% and 10 wt.% of nanocellulose reduced the water absorption and degradation of the films by approximately 50% after 130 min of exposition. The same experiment was conducted in saline water, and lower absorption levels were observed in saline water than in distilled water for both unreinforced and reinforced films. The addition of 5 wt.% and 10 wt.% of CNC reduced swelling by forming an interconnected network with the matrix. Therefore, the absorption of saline water was restricted. The addition of crystalline nanocellulose to PVA increased water resistance, making them ideal for food packaging. However, also, after a short period of exposition, the CNC–PVA started to degrade, which is a positive attribute of sustainable packaging [69].

On the other hand, Zhang et al. [23] reported contrary results about the water absorption of CNC from wastepaper–PLA composites. Pristine PLA and composites prepared with 1–5 wt.% of CNC were soaked in water over seven weeks. The results indicated that the water absorption increased over time with the addition of nanocellulose. PLA is a hydrophobic material, whereas nanocellulose has a high content of hydroxyl groups with a strong affinity for water. Therefore, these results suggested that as the amount of PLA was reduced by the addition of CNC, the composite became less hydrophobic. Results may also indicate poor interaction between the nanocellulose and PLA.

### 4.6. Optical Properties

Cellulose nanocomposites have promising applications in packaging, where transparency and appearance are essential properties to be considered. Pires et al. [21] assessed the color, light transmittance (λ = 190–900 nm), and absorption (λ = 600 nm) of chitosan films reinforced with micro and nanocellulose using a calorimeter and an ultraviolet-visible (UV-Vis) spectrophotometer, respectively. Results showed that all composites exhibited a yellowish color, which became more intense with the addition of 2.5 wt.% reinforcement. Moreover, light absorption increased proportionally with the addition of nanocellulose and microcellulose, indicating that films became more opaque. This trend was confirmed by Srivastava et al. [52] and Yudhanto et al. [22,52], who reported that the increased concentration of nanocellulose creates a physical barrier to light. Furthermore, the UV-light transmittance of chitosan films has also been investigated [21,47], with results showing that chitosan films reinforced with nanocellulose have lower light transmittance compared to neat chitosan. Even though the incorporation of crystalline cellulose decreased the transparency of the films, the presence of the reinforcement originated an ultraviolet-visible light barrier. Sánchez-Gutiérrez et al. [24] calculated the UV barrier of CNF–PVA composites by measuring the UV transmittance in the 200–800 nm range. Results showed that adding 7.5 wt.% of CNF increased the UV barrier from 5.7% (neat PVA) to 48.8%. This is a desirable property for food packaging applications, as the films could prevent nourishment degradation.

Csiszár et al. [64] also found a decrease in transparency in thermoplastic starch films with increasing crystalline nanocellulose. However, they highlighted that the reduction in transparency was not noticeable to the human eye when incorporating 5–50 wt.% of CNC. Furthermore, the haze index of the composites was reported as a function of the reinforcement and plasticizer content, with results showing that the haziness of films was directly proportional to CNC content and inversely proportional to the plasticizer concentration. It was concluded that the scattering of the nanocellulose in the matrix plays an essential role in the optical properties of the composites. Adding a higher amount of plasticizers and lower reinforcement caused a well-dispersed composite due to reduced agglomeration, which helps obtain more transparent films. Correspondingly, Hamid et al. [45] developed chitosan composites based on pea pod CNC and reported that adding 10 wt.% of CNC reduced UV transmittance to 64% from 89% (neat chitosan) at λ = 700 nm. The authors explained that the agglomeration of cellulose observed at high concentration causes light diffusion, reducing film transparency. Despite the decrease in transparency, the cellulose-reinforced nanocomposites still provide a clear and transparent option as a replacement for traditional packaging [45,47].

### 4.7. Degradability

Biobased composites are sustainable alternatives to petrochemical-based polymers since biopolymers and biodegradable materials are used to develop these nanocomposites. Then, it is relevant to assess the biodegradability of these materials. Few studies have recently included a biodegradability analysis of nanocellulose biobased composites. Hamid et al. [45] developed chitosan-based composites reinforced with different concentrations of CNC derived from pea pods and assessed the soil burial degradability. The test was performed using the conditions established in the ISO 846 standard [79]. Degradation was measured at 7, 50, and 150 days. Results were illustrated as a function of weight loss percentage, which increased with time. Pristine chitosan exhibited about 19% of weight loss after 150 days of soil burial, which was the highest weight loss compared to composites reinforced with 1–10 wt.% of CNC. It was concluded that since chitosan is a hydrophobic material, moisture from the soil was the main factor for film degradation. Therefore, when nanocellulose was added, the network formed with the matrix reduced the penetration of water within the composite. Then, the composite became more resistant to soil degradation. On the other hand, Oyeoka et al. [69] reported that the addition of CNC to a PVA–gelatin matrix did not significantly affect soil burial degradation. After 28 days, the weight loss of the neat PVA–gelatin film, 5 wt.%, and 10 wt.% CNC/PVA–gelatin films were 12.90%, 12.58%, and 12.62%, respectively. Furthermore, the curves of weight loss percentage vs. time of all the films tested exhibited a continued and rapid degradation, which suggested that these materials possess a good ability to degrade. Different results were described by Kesari et al. [62], who investigated the biodegradability of thermoplastic starch films reinforced with different CNF loads (1, 3, and 5 wt.%). The burial test was performed for 60 days, and the weight change was measured. As the nanocellulose concentration increased, the weight loss percentage increased over the period tested. Composites with 5 wt.% of CNF exhibited 13% more weight loss than neat chitosan, showing its potential as an eco-friendly material. 

## 5. Surface Modification of Nanocellulose to Prepare Biobased Composites

It has been demonstrated that dispersion of the reinforcement in the matrix is an influential factor that tailors the final properties of nanocellulose composites. Nanocellulose tends to self-agglomerate during the drying processes since the interaction between hydroxyl groups of the nanocellulose surface is through hydrogen bonding. This process is called “hornification” and causes irreversible nanocellulose agglomeration [57,80]. It is so that different approaches have been reported to promote a better dispersion of the nanocellulose in the biopolymer matrix.

One strategy that has been explored is the impact of nanocellulose size on the interaction between polymer and matrix. This is because different nanocellulose lengths result in different surface charges and restrictions on polymer mobility, leading to varying interactions between the two. For instance, Wang et al. [81] developed PVA films reinforced with CNF prepared by TEMPO-mediated oxidation (TEMPO-CNF) of different dimensions. They assessed the effect of nanocellulose size on the microstructure and mechanical properties of the composites. The size of TEMPO-CNF was controlled by the amount of sodium hypochlorite (NaOCl) added, which resulted in a change in the length of the TEMPO-CNF from the micro- to the nanoscale. The mechanical properties of composites reinforced with different cellulose concentrations and lengths were reported. It was observed that incorporating up to 1.5 wt.% of cellulose enhanced tensile strength and Young’s modulus. Above this concentration, the mechanical properties slightly declined due to agglomeration, however, only for composites reinforced with nanoscale cellulose. However, for composites reinforced with more non-nanoscale cellulose, a gradual decline in mechanical properties was observed with increasing cellulose content. This suggested that the higher length of nanocellulose hampered the dispersion and interaction between the PVA and cellulose, and mechanical properties were weakened. Pavalaydon et al. [26] also assessed the effect of nanocellulose particle size on mechanical properties. They used PVA-based composites reinforced with CNC extracted from coir fiber and sugarcane bagasse, which possessed different particle sizes (137.3 nm and 48 µm, respectively) and crystallinity (crystallinity index of 1.03 and 0.85, respectively) after being treated under the same conditions [26]. The tensile strength of nanocellulose obtained from coir fiber was 63% higher than that obtained from bagasse when 0.5 wt.% of CNC was added. This suggested that the reduced particle size and higher crystallinity degree exhibited by CNC obtained from coir fibers promoted better dispersion and stronger interaction between the matrix and reinforcement. FTIR spectra results also indicated that the nanocellulose derived from coir fibers had higher hydroxyl (OH) concentrations, which contributed to a stronger interaction between the nanocellulose and PVA.

Another study compared the effect of the aspect ratio of different types of nanocellulose in the properties of poly(β-hydroxybutyrate) (PHB) based composites [27]. Composites were prepared using crystalline nanocellulose (CNC, aspect ratio = 8), nanocellulose fibers (CNF, aspect ratio = 70), and bacterial cellulose (BC, aspect ratio = 600) by solvent casting. SEM images revealed that the final dispersion of nanocellulose was determined by its flexibility, which was dependent on its aspect ratio. Crystalline nanocellulose demonstrated a rigid behavior due to its small aspect ratio, appearing as individual rods well dispersed in the biopolymer matrix. However, increasing the filler aspect ratio, rigidity decreased, and cellulose became more flexible. BC formed self-entanglements and clusters in the PHB matrix of its large aspect ratio and increased flexibility. The mechanical and viscoelastic properties demonstrated a strong relationship with the flexibility of nanocellulose. For example, the incorporation of 0.5 wt.% of CNC and CNF in PHB enhanced tensile strength by 23% and 10%, respectively, since their rigidity feature allowed a homogeneous dispersion. In contrast, BC promoted the formation of clusters and did not reinforce the biopolymer matrix. Furthermore, rheological results confirmed that BC self-entangled, restricting polymer mobility due to its high flexibility. As a result, BC–PHB composites showed the longest relaxation time compared to neat PHB, CNF, and CNC composites. Similarly, Ghalehno and Yousefi [28] prepared carboxymethyl cellulose (CMC) composites reinforced with four types of cellulose that had different morphology, dimensions, and surface chemistry. The cellulose was obtained from wheat straw using acid hydrolysis, TEMPO-oxidation, and mechanical methods to produce cellulose crystal (CNC), TEMPO-cellulose nanofiber (TCNF), mechanical (MCNF), and lignocellulose (LCNF) nanofiber, respectively. SEM images showed that TCNF was the thinnest nanomaterial, while MCNF and LCNF were the opposite. In the same way, TCNF and mechanically treated fibers exhibited the highest and lowest specific surface area, respectively. The cellulose’s dimensions and specific surface area affected its interaction with the matrix. Hence, it was expected that nanoscale cellulose with a high specific surface area would form a strong interface with CMC and result in an effective stress transfer. It is so that TCNF–CMC composites exhibited the highest tensile strength, Young’s modulus, and strain at the break at all reinforcement concentrations tested (1, 3, and 6 wt.%) since this type of nanocellulose had high specific surface and small dimensions.

Many studies [57,80,82,83,84,85] have reduced nanocellulose agglomeration and strengthened interfacial adhesion by surface chemical modification, which usually converts hydrophilic nanocellulose into hydrophobic to improve its dispersion in hydrophobic matrices. Some strategies to modify the surface of nanocellulose include using surfactants and coatings, oxidation, esterification, etherification, silane treatment, acetylation, and polymer grafting.

Nanocellulose modifications utilizing surfactants and coatings have been employed to prepare biobased composites. In a study by Lamm et al. [57] cellulose nanofibrils (CNF) were functionalized with chitosan (CS) to form strong interfaces and improve the dispersion of CNF–polylactic acid (PLA) composites. The functionalized CNF was prepared using a coprecipitation method with sodium hydroxide (NaOH). The optimum load of CS in CNF was found to be 2.5 wt.% with respect to the CNF content (CNF/2.5CS) since the nanocomposite exhibited the best mechanical properties at this load. Moreover, PLA composites were prepared at different CNF–2.5CS concentrations by melt processing, and mechanical and thermo-mechanical properties were compared with CNF–PLA composites. Young’s modulus and storage modulus increased with increasing the reinforcement content. However, a higher enhancement was provided when using functionalized nanocellulose. The authors proposed that, in addition to hydrogen bonding between PLA, CNF, and CS, covalent amide bonds were formed between CS and PLA during melt processing, resulting in a stronger interface between the matrix and filler. Nasution et al. [86] reported chitosan blends with PLA through a substitution reaction with phthalic anhydride to improve miscibility with fibril nanocellulose. Since chitosan is amphiphilic, it acts as a compatibilizer between the CNF and PLA. Therefore, enhanced miscibility of the nanocellulose was observed in SEM images of fractured composites. Another work reported the preparation and characterization of CNC/PLA composites utilizing crystalline nanocellulose, which was surface modified with tannic acid and coated with octadecyl amine (ODA) [87]. The modification increased the hydrophobicity of nanocellulose, as demonstrated by the increased contact angle from 20° (unmodified CNC) to 70° (modified CNC). Nevertheless, it was encountered that the addition of 2 wt.% of functionalized CNC did not increase tensile strength and elastic modulus compared to composites with 1 wt.% of functionalized CNC. The authors suggested that the mechanical properties were diminished due to agglomeration of the nanocellulose during melt processing, despite its surface modification.

The acetylation of nanocellulose is a more efficient strategy to avoid nanocellulose self-aggregation. As an example, Xu et al. [82] developed nanocellulose composites by solvent casting and used poly (3-hydroxybutyrate-co-3-hydroxy hexanoate) (PHBH) and acetylated cellulose nanocrystals (ACNs) as a matrix and reinforcement, respectively. The PHBH composite with 1 wt.% of CNC showed a rough surface due to agglomeration. On the other hand, PHBH composite with 1, 1.5, 2, and 2.5 wt.% of ACN exhibited a well-dispersed reinforcement due to the high compatibility between the modified nanocellulose and the biopolymer. The thermal stability, tensile strength, and Young’s modulus of ACN/PHBH composites were enhanced compared with CNC/PHBH composites. The results demonstrated that a well-dispersed phase promotes hydrogen bonding and an efficient stress transfer through the interface of the composite. These findings are consistent with the study of Ren et al. [88] who reported PLA composites reinforced with acetylated CNF obtained through melt compounding. TEM images illustrated the homogeneous dispersion of 2 wt.% of modified CNF in the PLA matrix and the formation of a fibrillar network structure. Contrarily, nanocellulose agglomeration was observed in the CNF–PLA composite due to the incompatibility between the hydrophilic reinforcement and hydrophobic biopolymer.

Silane treatment is another approach to enhance dispersion, in which nanocellulose is modified with a silane agent to form covalent bonds with the biopolymer, thereby improving compatibility and distribution. Oyekanmi et al. [83] developed macroalgae films reinforced with modified fibril nanocellulose and assessed the effect of the silane treatment modification on the composite properties. A higher gain in tensile strength was observed for composites prepared with modified CNF than those with an unmodified reinforcement, which suggested that the surface modification helped to improve the dispersion and interaction of the nanocellulose with the macroalgae. These results were confirmed by SEM images, in which a dense and free-of-agglomeration structure was observed when up to 4 wt.% of treated reinforcement was added to the biopolymer. As the nanocellulose loading increased, tensile strength and stiffness improved, with a higher reinforcement effect observed for composites made with silane-modified CNF. The authors suggested that the formation of crosslinking sites through Si-O-Si bonds enhanced mechanical properties and that thermal stability and wettability were improved by a strong molecular interaction between the hydroxyl and silane groups of the biopolymer and the modified CNF, respectively.

A different approach to improve the interaction between nanocellulose and chitosan matrix is the surface modification of nanocellulose through oxidation by periodate [84]. This reaction forms aldehyde terminations in the nanocellulose, which later reacted with the amino groups of chitosan, enhancing compatibility between matrix and reinforcement. Gao et al. [84] reported the modification of CNC using sodium periodate to form dialdehyde nanocrystalline cellulose (DANC), which was then used to prepare chitosan composites by solvent casting. Mechanical testing showed that increasing the DANC concentration from 0–50 wt.% increased tensile strength from 23.06 MPa to 41.06 MPa, respectively. High reinforcement loads were successfully incorporated into chitosan due to the Schiff base reaction between DANC and chitosan, which promoted compatibility and an efficient stress transfer through the interface. A further effect of the enhanced compatibility caused by the surface modification of cellulose was evidenced in the gas barrier properties of the composites. The addition of 25% of modified nanocellulose decreased the water vapor transmission rate (WVTR) by approximately 44%, while the addition of unmodified CNC decreased WVTR by 23%. It was suggested that two main factors contributed to this dramatic reduction. First, the restriction in the moisture diffusion was due to enhanced interaction between the nanocellulose and biopolymer. Second, the crosslinking network formed between DANC and chitosan.

Nanocellulose surface modification through esterification reaction has shown to be one of the most promising techniques. Esterification modification is carried out with the reaction between the hydroxyl groups of nanocellulose and acids or anhydrides. Szefer et al. [80] reported the surface modification of nanocellulose with succinic anhydride (SA) under microwave irradiation, to then use the modified nanocellulose to prepare PLA composite by melt processing. FTIR analysis of modified nanocellulose showed a new peak absorption at 1725 cm^−1^, corresponding to carbonyl groups formed due to the esterification reaction. SEM results revealed reduced or even nonsignals of nanocellulose agglomeration at 1 wt.% and 3 wt.% of reinforcement. These results were then associated with the higher glass transition and storage modulus of the composites prepared with modified nanocellulose due to the enhanced dispersion of nanocellulose in the PLA matrix. Furthermore, Lee et al. [89] reported that PHBH composites prepared with CNF modified through esterification exhibited, not only improved mechanical, thermal, and rheological properties but also enhanced processability. Nanocellulose was surface modified with alkenyl succinic anhydride (ASA) and then mixed with PHBH using a twin-screw extruder at different reinforcement concentrations (5, 10, 14, and 17 wt.%). In this study, the dispersion of the modified CNF in the biopolymer was assessed by X-ray computed tomography (CT). These images showed that nanocellulose is well distributed, forming a network structure with the matrix. No evidence of agglomeration was observed even at high reinforcement loads. Then, good compatibility between the modified CNF and PHBH was associated with the improved properties of the biobased composites. For example, CNF–PHBH composites prepared with 10 wt.% of modified nanocellulose exhibited approximately 71%, 26%, and 17% higher tensile strength, Young’s modulus, and elongation at break than neat PHBH, respectively. This demonstrates that esterification is an effective strategy to incorporate high concentrations of nanocellulose, which has not been accomplished by any other mechanisms herein reported. Moreover, the molecular weight reduction of PHBH was assessed after melt processing by a high-performance liquid chromatography (HPLC) system. Although the molecular weight of PHBH was reduced after melt processing, the reinforcement effect of CNF compensated for this reduction. Enhanced mechanical properties were observed with the increase of the reinforcement concentration.

A different approach to overcome the incompatibility between nanocellulose and biopolymers is through the modification of the matrix via cross linking. For example, Chin et al. [70] modified PVA through crosslinking reactions with ethane dioic acid (EA) to prepare composites with CNC. The mechanical properties of the crosslinked composites were compared to noncrosslinked composites at different reinforcement concentrations (1, 3, 5, and 7 wt.%). A 105% increase in tensile strength was observed for the crosslinked composites containing 3 wt.% of CNC, compared to only a 60% increase in noncrosslinked composites. The good compatibility tailored by the crosslinking modification promoted good dispersion of nanocellulose in PVA and created a stronger interface, which led to a positive increment in tensile strength and stiffness. However, a decrease in tensile strength and Young’s modulus was observed for both composites when the reinforcement concentration exceeded 3 wt.%. The reduced availability of hydroxyl groups contributes to the agglomeration of CNC when there is an excess of reinforcement, which then induces weak points in the composite. Moreover, the decomposition temperature increased, and the WVP decreased in crosslinked composites, demonstrating a 3D crosslinked network formation through esterification. Zhao et al. [90] found similar results and observed improvement in the water barrier and mechanical properties of agar-based composites prepared with calcium chloride and CNC as cross-linking agents and reinforcement, respectively.

Polymer grafting is also suitable for improving compatibility between nanocellulose and hydrophobic biopolymers. This process can be achieved through in situ reactive extrusion, where compatibilization occurs under the melt state. Li et al. [85] reported the grafting of PLA onto the nanocellulose fibers (CNF-g-PLA). The composites were prepared with in situ reactive extrusion using dicumyl peroxide (DCP) as the radical initiator. A comparison of mechanical properties between 5 wt.% CNF–PLA and 5 wt.% CNF-g-PLA at different DCP concentrations was reported. CNF-g-PLA composites increased in tensile strength, tensile modulus, and elongation at the break by 20%, 31.8%, and 12% compared to CNF–PLA, respectively. The reinforced effect observed in grafted composites was attributed to the improved interfacial adhesion and formation of a branched network, which led to an effective stress transfer mechanism. The concentration of DCP also affected mechanical properties, with higher concentrations leading to longer polymer chains and improved mechanical performance. These results agreed with the DMA characterization, which revealed increased interfacial adhesion obtained with grafting copolymerization. CNF-g-PLA initiated with 0.75 parts per hundred rubbers (phr) of DCP exhibited a storage modulus (E’) of 3610 MPa compared with the CNF–PLA that showed an E’ of only 3221 MPa at 30 °C. The interfacial adhesion was calculated from DMA data by the adhesion factor (A). Stronger interaction will result in a smaller adhesion factor. CNF–PLA composites exhibited an A value of 0.12, while grafted composites displayed A values between 0.01–0.03. SEM images showed enhanced compatibility and dispersion in CNF–g–PLA composites, and the formation of PLA chain entanglements in grafted composites was also observed.

As an alternative mechanism to improve the compatibility between PLA and nanocellulose, Shazleen et al. [91] posited the grafting polymerization of PLA with maleic anhydride (MA) (PLA–g–MA) to prepare CNF–PLA composites by reactive extrusion. Composites were prepared at 10 wt.% of MA and 0.75 wt.% of dibenzoyl peroxide (DBPO), where MA acted as the compatibilizer and DBPO as the initiator. Moreover, composites were formed by neat PLA, PLA–g–MA, and 3 wt.% of CNF. It was reported that the addition of PLA–g–MA to the composite increased the rigidity of the composite due to mobility restrictions caused by the grafting between the PLA and the anhydride. With the addition of 3% wt. of PLA–g–MA, Young’s modulus increased from 2.9 GPa (neat PLA) to 11.7 GPA. Contrary to the results presented by Li et al. [85] (discussed above), the tensile strength of composites with PLA–g–MA gradually decreased with the addition of the grafted polymer, and the values were slightly lower than CNF–PLA composites [85]. This observation was attributed to the plasticizer effect of PLA–g–MA, which was confirmed with a reduction in T_g_ [91].

## 6. Environmental Impact on the Preparation of Nanocellulose and Composites

**Figure 3:**
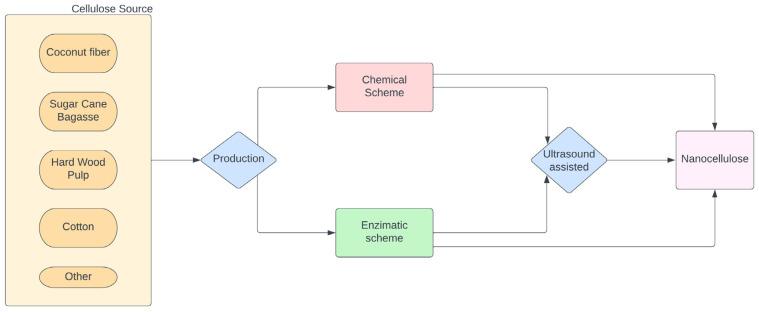
System boundary of cradle-to-gate production of nanocellulose.

The environmental impact analysis of products draws great importance as sustainability is essential in developing greener alternatives. Climate change affects the life of every living being. Different methodologies exist to assess the environmental burden of materials, products, or services. Life cycle assessment (LCA) is a tool based on circular economy theory as it quantifies the environmental impact of a product’s life stages [29]. These stages may include the impacts related to the extraction of raw materials, processing or manufacturing, the use of the product, and the end-of-life scenarios. For this review, the cradle-to-gate scenario is considered as the use and disposal may vary depending on the application given to the nanocellulose. The LCA of different nanocellulose production schemes and raw materials have been assessed in this section. Global warming (kg pf CO_2_-eq), terrestrial acidification (kg of SO_2_), eutrophication (kg of P), and other impact indicators can be evaluated to find out the most effective (in terms of environmental impact) production route for nanocellulose. Figure 3 shows the schematic representation of the life cycle for nanocellulose production from different raw materials. The scheme shows cradle-to-gate life cycle analysis.

There are different sources, pathways, and products in the nanocellulose family. These sources are dependent on natural resources that contain high amounts of cellulose. Nanocellulose preparation paths are divided into two main groups, chemical and enzymatic. Nanocellulose is found as crystals (CNC), fibrils (CNF), yarn, whiskers, lignin-containing cellulose nanocrystals (LCNCs), acetylated cellulose nanofibrils (AcCNF), and other names given to the nanometric scale and form of the obtained cellulose. Table 3 shows the papers analyzed in the current review that contain life cycle assessment perspectives on different sources, production schemes, and types of nanocellulose–polylactic acid (PLA) composites as examples of nanocellulose applications. It also includes some environmental indicators evaluated in the studies. One review [92] paper has gathered experimental routes to produce nanocellulose considering LCA methodology and presenting each route’s environmental impact. However, the review paper only gathered information until 2021, there is new research in this area that has been included in the current study.

Arvidsson et al. [93] conducted a cradle-to-gate life cycle assessment (LCA) of the production of cellulose nanofibrils using enzymatic and carboxymethylation pretreatment options. They focused on producing 1 kg of cellulose nanofibers and utilized both primary and secondary data to evaluate the environmental profile. Three different production routes from wood pulp are shown in Table 3. Climate change, terrestrial acidification, and water depletion were obtained from the evaluated scheme (Table 3). The carboxymethylation route showed a higher environmental impact across all categories. At the same time, the enzymatic and nontreatment processes had a similar impact in three categories. Also, energy demand for the functional unit was assessed in the study but has not been included in the review.

Piccino et al. [94,95] used carrot processing waste to produce nanocellulose fibers using two enzymatic-production methods, whether wet or electro-spinning. This study had a scale-up approach from a laboratory to an industrial production scale. The authors have considered ReCiPe [103] midpoint and endpoint methods using the Ecoinvent database through Open LCA [104] software. The study found no significant difference in the global warming potential of the two production processes.

In addition, one study [90] evaluated the production of cellulose nanocrystals from coconut husk, taking the ReCiPe [103] midpoint at a hierarchical level method into account using SimaPro [105] software. The use of ultrasound had a significant contribution towards climate change and on almost every indicator, followed by pulping. Even though it is not the most contaminated production scheme, it was important to consider how to improve environmental performance. Table 3 shows results for climate change, terrestrial acidification, freshwater eutrophication, marine eutrophication, human toxicity, and water consumption categories, which have been analyzed in the current review paper. In contrast, Turk et al. [97] analyzed the LCA of nanocellulose fibril production using a TEMPO (2,2,6,6-Tetramethylpiperidinyloxy) oxidation reaction. The authors studied the variation of results using different impact-assessing methods such as ILCD (International Reference Life Cycle Data System) [106], CML (Centrum voor Milieukunde Leiden) [107], and ReCiPe [103] at a midpoint level. Table 3 shows the environmental impact indicators only for the ReCiPe [103] midpoint method. It was concluded that the method is more precise with less uncertainty. In the global warming potential, the variation is minimal, resulting in around 800 kg of CO_2_ per functional unit. The variations are found in the assignment of characterization factors, i.e., methane produces more CO_2_ according to the recipe than CML [107].

Haroni et al. [92] evaluated the environmental profile of four routes for cellulose nanofiber production. A combination of organo-solvent (ethanol–water) and alkaline pretreatment was used in this study. Then TEMPO or lime juice was used to degrade the polymer chains. The study uses ILCD [106] (Table 3) and IPCC (Intergovernmental Panel on Climate Change) [108] methods to assess the environmental impact of the options considered. The alkaline lignin extraction and lime juice hydrolysis presented the lowest indicator regarding global warming potential, terrestrial acidification, and human toxicity. Meanwhile, the organo-solvent and lime juice processes had the highest indicators. The IPCC [108] results are not presented in Table 3, though they had a similar tendency to the results from ILCD [106]. The routes evaluated were an organo-solvent pretreatment, then a TEMPO oxidation, and a high-pressure homogenization, known as OTH. OLH uses lime juice instead of the last two processes in OTH. Similarly, ATH uses an alkaline pretreatment and the same processes as OTH. In contrast, ALH uses alkaline pretreatment and lime juice hydrolysis. The global warming potential for the processes (OTH, OLH, ATH, and ALH) are 8.92, 11.20, 4.45, and 3.17 kg of CO_2_ per functional unit, respectively. One study evaluated the life cycle of lignin-containing CNC (LCNC) production from thermomechanical pulp and CNCs without lignin [109]. TRACI (tool for reduction and assessment of chemicals and other environmental impacts) was used to evaluate global warming and acidification potentials. Additionally, cumulative energy demand was assessed, though not included, in Table 3. Regarding carbon emissions, CNC performed better than LCNC. The lignin-containing nanocellulose had about 30 kg of CO_2_ and 0.15 kg of SO_2_ per kg of produced cellulose. Methods and approaches affect the results proposed in the analyzed study.

Bondancia et al. [99] proposed a method for isolating cellulose nanocrystals from sugar cane bagasse using organic and inorganic solvents and used CML [107] in SimaPro [105]. Sugar cane bagasse is assumed to have no environmental impact as it is a waste from the sugar production industry. Furthermore, only global warming was evaluated in the study. The citric acid production process had the least impact, while the other two had almost 2.5 times more CO_2_ emissions. Another study that also used sugar cane bagasse as raw material focused on a multiproduct biorefinery [100]. This study used Impact 2002+ [110] as the impact-assessing method at the midpoint and endpoint levels. However, only the global warming potential has been analyzed. The biorefinery approach had a slight reduction in the impact category. Krexner et al. [101] assessed the production of nanofibrillated cellulose from the manure of elephants and compared it to hardwood chips. ReCiPe [103] midpoint H was used as the impact assessment method using OpenLCA for 1 kg cellulose nanofibers production. The results showed similar environmental profiles comparing manure to wood. Almost no significant variation can be established for the two production processes for that functional unit. However, for industrial or bulk production, 0.1 kg of CO_2_ eq is saved by using manure as a cellulose source and may become more relevant. Moreover, Zhang et al. [102] evaluated changing some chemical processes by adding mechanical steps to reduce the use of chemicals in the CNCs production process. The results of this study showed a significant reduction in the environmental impact categories when microfiltration is included after the acid hydrolysis. A 1.6 times reduction was estimated for carbon emissions between the different routes using the Impact 2002+ [109] cycle impact assessment method. Across all the categories, one of the routes (acid hydrolysis + microfiltration + disc stack centrifugation) presented a better environmental profile due to the added separation processes to reduce the use of chemicals and water used to neutralize the acid in the hydrolysis.

Two critical studies in the literature focus on the cradle-to-gate life cycle of nanocellulose composites. Hervy et al. [102] used nanocellulose produced from bacteria and fiber from chemical preparation to be used as filler in epoxy composites. The composite using bacterial nanocellulose resulted in higher global warming than the regular nanofiber composite using CML 2001 [107] method using GaBi [110] software, version 6.0. Compared to polypropylene or polylactic acid (PLA) composite, those alternatives had better performance in carbon emissions. Additionally, Kanematsu et al. [98] analyzed the greenhouse gas emissions of PLA–acetylated nanofibrils cellulose composite. The authors used the IPCC [108] to estimate the emission of greenhouse gases. The study divided the process into different cradle-to-gate assessments considering the acetylation process, fabrication of acetylated nanofibers out of kraft pulp, and the preparation of the PLA composite. High and low uncertainty-based assessments were made for composite preparation at both laboratory and industrial scales. Global warming impacts are 10–13 kg and 7–10 kg CO_2_ eq for lab production and the industrial scale composite preparations, respectively.

Further studies should be done on composite preparations containing nanocellulose in crystal, fiber, or yarn forms. The environmental impact of the actual product should be evaluated by comparing different options with the same applications. Besides, the use and disposal of biocomposites must be assessed to evaluate the reduction of an environmental impact compared to synthetic nondegradable polymer composites.

## 7. Conclusions

The incorporation of nanocellulose into biopolymer has been extensively studied in the literature, varying the raw materials from which nanocellulose is synthesized. The impact of the amount of nanocellulose as the filler on the compatibility with different synthetic and natural polymeric matrices has been assessed. This review paper analyzed the literature and advances in biopolymer composites that implement nanocellulose. It gathers the results from the effect of processing and modifications on the mechanical, optical, physical, chemical, and oxygen and water vapor barrier properties. The study also includes examining the environmental impact of different production methods and nanocellulose composites, providing insights into the sustainability of this alternative to petroleum-based synthetic polymers.

Polymer composites that use nanocellulose improve these biomaterials’ mechanical, thermal, and barrier properties. Nanocellulose biobased composites are mainly prepared by solvent casting and melt processing. Regardless of the composite preparation method, achieving good dispersion of the nanocellulose in the matrix is essential to maximize the reinforcement effect. However, nanocellulose tends to form agglomerates when added to a polymer matrix, diminishing the properties of the biocomposites. Stirring and ultrasonication steps must be involved in solvent-casting processes to improve the dispersion of nanocellulose. Furthermore, melt processing of nanocellulose composites has proved to be an adequate method to prepare composites with additives and introduce some valuable properties to the composites. However, the effect of the shear forces may cause degradation of the biopolymers during melt processing. The processing technique, the compatibilizer, and the low nanocellulose content result in a composite with better filler and biopolymer interactions. In addition, chemical modifications such as acetylation, cross linking, and grafting are some of the mechanisms that improve mechanical strength, thermal resistance, and nanocellulose dispersion into the matrix. Moreover, the biocomposites’ permeability to oxygen and water vapor decreases with the addition of nanocellulose because the interaction between nanocellulose and matrix through hydrogen bonds forms a tortuous path that restricts water vapor and oxygen diffusion. LCA results indicate that the environmental impact of nanocellulose is mainly dependent on the production path and the raw material used, with the enzymatic path being more environmentally friendly and having a lower global warming potential.

## Figures and Tables

**Table 2:** Melt-processing conditions of nanocellulose biobased composites.

Reinforcement (Source)	Matrix	Processing Conditions	Mechanical Properties		
			Tensile Strength (MPa)	Young’s Modulus (GPa)	Elongation (%)
**CNC** **(sugar palm)** [54]	**PLA-Sugar palm starch**	Solvent: distilled water NC content: 0.5 wt.% Plasticizer: 15 wt.% glycerol and 15 wt.% sorbitol Mechanical stirring for 30–45 min at 80 °C Melt blending at 170 °C for 13 min and 50 rpm Hot processing	-	-	-
CNF (bamboo pulp) and corn starch [61]	PVA	NC content: 0, 10, and 20 wt.% Plasticizer: Formamide and water Melt mixed at 150 °C, 30 rpm for 10 min Compression molding	19.5–28.1	0.7–1.6	2.5–11.0
CNF (cardboard) [56]	PLA	NC content: 0–40 wt.% Melt mixed in a Brabender at 170 °C, 60 rpm for 3–5 min Compression molding at 175 °C	52.0–60.0	3.4–5.9	-
CNF [55]	PLA	NC content: 0–6 wt.% Melt blended in a Brabender at 170 °C, 30 rpm for 30 min Compression molding at 160 °C for 10 min.	68.5–76.1	2.9–3.3	-
CNF/CS [57]	PLA	CNF–CS content: 5–30 wt.% Meltblended in a Brabender 175 °C, 60 rpm for 3 min Compression molding at 180 °C	51.0–58.0	3.0–4.2	-
CNC (bamboo) [59]	PLA/PBS	NC content: 0–1.5 wt.% 20 wt.% PBS Melt mixed at 175 °C and 60 rpm for 10 min Compression molding at 180 °C and 150 MPa for 4 min Postcuring at 50 °C for 24 h	69.0–86.0	8.3–10.4	12.0–18.0
LCNF (palm waste) [60]	PLA	NC content: 0–10 wt.% Plasticizer: 20 wt.% PEG Mechanical stirring and sonication at 30 °C Melt compounding in a corotating conical twin-screw at 190 °C and 100 rpm for 5 min	12.5–20.0	0.4–1.4	2.0–100.0
CNF (bamboo) [62]	Corn starch	NC content: 0–5 wt.% Plasticizer: 30 wt.% glycerol and 1 wt.% stearic acid. Melt processing in a co-rotating twin-screw extruder from 90–130 °C. Compression molding at 70 MPa and 130 °C for 5 min.	3.0–5.1	0.9–2.1	22.2–48.6

**Table 3:** Literature review for the Life Cycle Assessment of the production of nanocellulose and its composites.

Research/Type of Nanocellulose	Production Scheme	Source	GWP (kg CO_2_ eq)	TA (kg SO_2_ eq)	FE (kg P eq)	ME (kg N eq)	HT (kg 1,4-DB eq)	WD (m^3^)
Arvidsson et al. [93]/CNF	Enzymatic pretreatment	Wood Pulp	0–2	0–0.02	-	-	-	0.2–0.3
	Carboxymethylation pretreatment		95–105	0.17–0.2	-	-	-	1–1.1
	No pretreatment		0–5	0–0.02	-	-	-	0.1–0.2
Piccino et al. [94,95]/CNF	Enzymatic-Wet Spinning	Carrot waste	1.9	-	-	-	-	-
	Enzymatic-Electro Spinning		1.6	-	-	-	-	-
Hervy et al. [96]/CNF-Epoxy composite	Bacterial Cellulose	Kraft Pulp	14	-	-	-	-	-
	Nanofribillated Cellulose		8.5	-	-	-	-	-
do Nascimento et al. [90]/CNC	Oxidation-Ultrasound-Hydrolysis	Coconut Fiber	207	0.45	0.0568	0.0303	47.7	2.3
Turk et al. [97]/CNF	Tempo	Woody biomass	806.92	6.44	0.76	0.05	49.38	28.2
Kanematsu et al. [98]/AcCNF-PLA	Acetylation-kneading	Woody chips	5.20–11.20	-	-	-	-	-
Haroni et al. [92]/CNF	Organo-solvent/TEMPO/High-Pressure (OTH)	Sugarcane trash	7.89	0.07	-	-	61.37	-
	Organo-solvent/Lime Juice/High-Pressure (OLH)		9.96	0.09	-	-	85.23	-
	Alkaline/TEMPO/High-Pressure (ATH)		4.08	0.03	-	-	41.44	-
	Alkaline/Lime Juice/High-Pressure (ALH)		2.85	0.02	-	-	23.52	-
Zargar et al. [31]/LCNC-CNC	Deep Eutetic Solvent	Thermomechanical pulp	31.7	0.14	-	-	-	-
	Acid Hydrolysis		12.9	0.29	-	-	-	-
Bondancia et al. [99]/CNC	Citric acid	Sugar Cane Bagasse	389	-	-	-	-	-
	Sulfuric acid		879	-	-	-	-	-
	Citric + sulfuric acid		894	-	-	-	-	-
Katakojwala et al. [100]/NCC	Baseline	Sugar Cane Bagasse	286	-	-	-	-	-
	Biorefinery		213	-	-	-	-	-
Krexner et al. [101]/CNF	Fermentation-Grinding	Manure	1.4	0.01	0.0009	-	1.9	-
	Fermentation-Grinding	Hardwood chips	1.5	0.06	0.0008	-	2.1	-
Zhang et al. [102]/CNC	Acid hydrolysis + gravity settling	Cotton	45–50	0.35–0.4	0.014–0.016	-	-	-
	Acid hydrolysis + gravity settling + disc stack centrifugation		40–45	0.22–0.26	0.007–0.009	-	-	-
	Acid hydrolysis + centrifugation + disc stack centrifugation		40–45	0.25–0.3	0.008–0.009	-	-	-
	Acid hydrolysis + microfiltration + disc stack centrifugation		28–32	0.18–0.2	0.006–0.008	-	-	-

## Data Availability

Not applicable.

## Abbreviations

**Abbreviation**	**Definition**
A	Adhesion factor
AcCNF	Acetylated cellulose nanofibrils
ACNs	acetylated cellulose nanocrystals
AFM	Atomic force microscopy
ALH	Alkaline/Lime Juice/High-Pressure treatment
ASA	Alkenyl succinic anhydride
ATH	Alkaline/TEMPO/High-Pressure treatment
BC	Bacterial nanocellulose
CMC	Carboxymethyl cellulose
CML	Centrum voor Milieukunde Leiden
CNC	Cellulose nanocrystals
CNF	Cellulose nanofibrils
CNF-g-PLA	PLA grafted onto the nanocellulose fibers
CS	Chitosan
CT	X-ray computed tomography
DANC	Dialdehyde nanocrystalline cellulose
DCP	Dicumyl peroxide
DMA	Dynamic Mechanical Analysis
DOE	Department of Energy
DSC	Differential scanning calorimetry
DTG	Derivative thermogravimetric analysis
E’	Tensile storage modulus
EA	Ethane dioic acid
FE	Freshwater eutrophication category
FITR	Fourier Transform Infrared Spectroscopy
GWP	Global warming potential category
HPLC	High-Performance Liquid Chromatography
HT	Human toxicity category
ILCD	International Reference Life Cycle Data System
IPCC	Intergovernmental Panel on Climate Change
LCA	Life cycle assessment
LCNF	Lignin-containing cellulose fibrils
MA	Maleic anhydride
MCNF	Mechanical cellulose nanofiber
ME	Marine eutrophication category
MFI	Melt flow index
NaOCl	Sodium hypochlorite
NaOH	Sodium hydroxide
NC	Nanocellulose
ODA	Octadecyl amine
OLH	Organo-solvent/Lime Juice/High-Pressure treatment
OP	Oxygen permeation
OTH	Organo-solvent/TEMPO/High-Pressure treatment
OTR	Oxygen transmission rate
OWP	Orange waste powder
PBS	Poly (butylene succinate)
PEG	Polyethylene glycol
PHB	Poly(β-hydroxybutyrate)
PHBH	Poly (3-hydroxybutyrate-co-3-hydroxyhexanoate)
PHMB	Polyhexamethylene biguanide
phr	parts per hundred rubbers
PLA	Polylactic acid
PLA–g–MA	PLA grafted with maleic anhydride
PVA	Polyvinyl alcohol
SA	Succinic anhydride
SEM	Scanning electron microscope
TA	Terrestrial acidification category
TCNF	TEMPO–cellulose nanofiber
TEM	Transmission electron microscopy
TEMPO	(2,2,6,6-Tetramethylpiperidinyloxy) mediated oxidation
TGA	Thermogravimetric analysis
TPS	Thermoplastic starch
TRACI	Tool for Reduction and Assessment of Chemicals and Other Environmental Impacts
UV-Vis	Ultraviolet-visible spectrophotometer
WD	Water consumption category
WVP	Water vapor permeability
WVTR	Water vapor transmission rate
XRD	X-ray Diffraction
tanδ	Loss tangent
T_c_	Temperature of crystallization
T_g_	Glass transition point
T_m_	Melting temperature
T_max_,	Temperature of maximum thermal degradation
T_onset_	Temperature of the initial thermal degradation
X_c_	Degree of crystallinity
ΔH	Heat capacity
